# Yap signalling regulates ductular reactions in mice with CRISPR/Cas9-induced glycogen storage disease type Ia

**DOI:** 10.1080/19768354.2022.2139755

**Published:** 2022-11-07

**Authors:** Yixia Xie, Baowei Hu, Yue Gao, Yaxin Tang, Guohe Chen, Jiayuan Shen, Zhikai Jiang, He Jiang, Jiwei Han, Junyan Yan, Lifang Jin

**Affiliations:** a School of Life Science, Shaoxing University, Shaoxing, Zhejiang, China; b Shaoxing Academy of Biomedicine of Zhejiang Sci-Tech University, Shaoxing, Zhejiang, China; c Department of Pathology, Affiliated Hospital of Shaoxing University, Shaoxing, Zhejiang, China; d The Second Clinical Medical College of Wenzhou Medical University, Wenzhou, Zhejiang, China; e The First Clinical Medical School of Zhejiang Chinese Medical University, Hangzhou, Zhejiang, China

**Keywords:** GSD-Ia, ductular reactions, yap, CRISPR-Cas9, *G6pc*

## Abstract

Glycogen storage disease type Ia (GSD-Ia) is caused by a deficiency in the glucose-6-phosphatase (G6Pase, G6pc) enzyme, which catalyses the final step of gluconeogenesis and glycogenolysis. Accumulation of G6pc can lead to an increase in glycogen and development of fatty liver. Ductular reactions refer to the proliferation of cholangiocytes and hepatic progenitors, which worsen fatty liver progress. To date, however, ductular reactions in GSD-Ia remain poorly understood. Here, we studied the development and potential underlying mechanism of ductular reactions in GSD-Ia in mice. We first generated GSD-Ia mice using CRISPR/Cas9 to target the exon 3 region of the *G6pc* gene. The typical GSD-Ia phenotype in *G6pc*^−/−^ mice was then analysed using biochemical and histological assays. Ductular reactions in *G6pc*^−/−^ mice were tested based on the expression of cholangiocytic markers cytokeratin 19 (CK19) and epithelial cell adhesion molecule (EpCAM). Yes-associated protein 1 (Yap) signalling activity was measured using western blot (WB) analysis and quantitative real-time polymerase chain reaction (qRT-PCR). Verteporfin was administered to the *G6pc*^−/−^ mice to inhibit Yap signalling. The CRISPR/Cas9 system efficiently generated *G6pc*^−/−^ mice, which exhibited typical GSD-Ia characteristics, including retarded growth, hypoglycaemia, and fatty liver disease. In addition, CK19- and EpCAM-positive cells as well as Yap signalling activity were increased in the livers of *G6pc*^−/−^ mice. However, verteporfin treatment ameliorated ductular reactions and decreased Yap signalling activity. This study not only improves our understanding of GSD-Ia pathophysiology, but also highlights the potential of novel therapeutic approaches for GSD-Ia such as drug targeting of ductular reactions.

## Introduction

Glycogen storage disease type Ia (GSD-Ia, MIM232200) is caused by deleterious mutations in the *G6pc* gene encoding glucose-6-phosphatase-α (G6Pcase-α, G6pc), which catalyses the hydrolysis of glucose-6-phosphate (G6P) into glucose during the terminal steps of gluconeogenesis and glycogenolysis (Shieh et al. 2002; Udawat et al. 2019). Mutations in *G6pc* can cause glycogen accumulation in certain organs and tissues, especially the liver and kidney, impairing their ability to function normally(Kishnani et al. 2014). Patients with GSD-Ia experience growth retardation, severe hypoglycaemia during fasting, and increased serum levels of uric acid, triglycerides, and cholesterol (Wang et al. 2013). Several animal models of GSD-Ia exist, including a GSD-Ia knockout mouse model (*G6pc*^−/−^) (Lei et al. 1996), a naturally occurring dog model (Kishnani et al. 2001), and two conditional G6pc-null mouse models (Peng et al. 2009; Mutel et al. 2011).

Ductular reactions are characterised by the proliferation of cholangiocytes and hepatic progenitors induced by liver injury (Sun et al. 2019; Sato et al. 2019b). Ductular reactions can be identified in various liver disorders including fatty liver disease (Richardson et al. 2007; Gadd et al. 2014). G6pcase-α (G6pc) deficiency can result in increased glycogen storage, leading to fatty liver disease in patients with GSD-Ia (Monteillet et al. 2018). To date, however, research on hepatic ductular reactions in GSD-Ia remains scarce. Ductular reactions area common pathology in the progression of various liver diseases, including liver fibrosis, cirrhosis, and cancer (Sato et al. 2019a). Therefore, studying ductular reactions in GSD-Ia may help reveal the mechanism underlying GSD-Ia-induced liver diseases, such as hepatocellular adenocarcinoma (HAC) and carcinoma (HCC), which are commonly found in adult GSD-Ia patients.

Traditional gene editing in animals is dependent on embryonic stem cells (ESCs) and homologous recombination techniques (Capecchi 1989). However, ESC-mediated gene editing is relatively time consuming and inefficient. Recently, the bacterial CRISPR/Cas9 system, which consists of Cas9 nuclease and single-guide RNA (sgRNA) targeting a gene of interest, has been applied for rapid genome editing in different species (Hwang et al. 2013; Wang et al. 2013). In this study, we used CRISPR/Cas9 technology to generate *G6pc*^−/−^ mice, which showed most known symptoms of human GSD-Ia, including ductular reactions. Furthermore, Yap signalling activity in the liver of GSD-Ia mice increased, while treatment with the Yap inhibitor verteporfin rescued ductular reactions and decreased Yap signalling activity.

## Materials and methods

### Animal use and care

The C57BL/6J mice were purchased from the Jackson Laboratory (Bar Harbour, Marine, USA). All mice were bred and maintained at an animal facility under specific pathogen-free conditions. Mice were kept under a 12 h-12 h light–dark cycle with food and water provided *ad libitum* from the cage lid. Both males and females were used, unless otherwise stated. All animal procedures were performed under the ethical guidelines of Shaoxing University (Shaoxing, Zhejiang, China) and conformed to the National Institutes of Health (NIH) guidelines.

### Production of Cas9 mRNA and sgRNA

A T7 promoter was added to the Cas9 coding region by polymerase chain reaction (PCR) amplification of px260 (Addgene, USA) using the primers CAS9-F (TAATACGACTCACTATAGGGAGATTTCAGGTTGGACCGGTG) and CAS9-R (GACGTCAGCGTTCGAATTGC). The T7-Cas9 PCR product was purified and used as a template for *in vitro* transcription (IVT) with a mMESSAGE mMACHINE T7 UltraKit (Life Technologies, USA).The T7 promoter was added to the sgRNA template by PCR amplification of px330 using primer pairs to target deletion of the exon 3 region in the *G6pc* gene (SgRNA1: AGAGATGAATGGACTTACGA; SgRNA2: CAAGGTGGAAGAACCCCTCT). The T7-sgRNA PCR product was purified- and used as the template for IVT with-aMEGA Short-Script T7 Kit (Life Technologies, USA). Cas9 mRNA and sgRNA were purified using a MEGA Clear Kit (Life Technologies, USA) and eluted in RNase-free water.

### Injection of Cas9 mRNA and sgRNA into mouse zygotes

*G6pc* (targeting exon 3) knockout mice were generated using CRISPR/Cas9, as described previously (Yen et al. 2014; Wang et al. 2017). Briefly, Cas9 mRNA and sgRNA were microinjected into fertilised embryos of C57BL/6J mice. The gene-edited embryos were cultured in modified potassium simplex optimised medium (KSOM) with amino acids (Millipore, USA) to the blastocyst stage at 37°C under 5% CO_2_ in air. The two-cell stage embryos were transferred into the oviduct of pseudo-pregnant ICR females at 0.5 days post coitum with 15–20 embryos per side. Recipient mothers delivered the pups naturally.

### Sanger sequencing analyses

The PCR products were amplified from mouse tissues or blastocysts and purified with a Universal DNA Purification Kit (Tiangen, China). An Illumina library was constructed using a NEBNextUltra DNA Library Prep Kit for Illumina (New England Biolabs, USA) as per the manufacturer’s instructions. Qualified libraries were applied to 2× 150-bp paired-end sequencing on the HiSeqX-ten platform (Illumina) at the Shanghai Biotechnology Corporation (China). Primers included forward: 5’-GAGTGGATAGTGAGATGGGTGGAT-3’; and reverse: 5'-ACAAGGGCTACCCTAGTGGAT-3’.

### Off-target analysis

Top off-target sites were predicted using the online CRISPR Design Tool developed by the Molecular Genome Engineering Lab, Hanyang University, Korea (http://www.rgenome.net/cas-offinder/). The PCR products for these potential off-target sites were subjected to T7E1 assay and Sanger sequencing.

### Phenotypic characterisation

In order to ensure *G6pc*^−/−^ mice had adequate milk supply, one female mouse was used to take care of 1∼2 *G6pc*^−/−^ mice. Mice ranging in age from 0 to 3 months were evaluated continuously regarding the pathophysiology of G6Pase deficiency. Measured parameters included weight, liver:body weight ratio, kidney:body weight ratio, and blood glucose, cholesterol, and triglycerides. Concentrations of glucose, cholesterol, and triglycerides were detected using automated clinical chemistry analysers (Hitachi, Japan) with corresponding detection kits (Roche, Switzerland). In the following study, we used around 3-week-old mice (18–22 days) for all experiments. There were 8∼10 mice in each group.

### Histological analysis

For histological observations, formalin-fixed liver tissues were embedded in paraffin and then cut into 5-μm thick sections. To detect liver injury, the samples were mounted on glass microscope slides and stained with haematoxylin & eosin (H&E), Sirius Red (Bestbio, China), and Periodic Acid Schiff (PAS) (Solarbio, China) for histological evaluation. Sirius Red and PAS staining protocols were performed according to the manufacturer’s instructions.

### Immunohistochemical analysis

The paraffin-embedded liver sections were consecutively immersed in xylene and ethanol for deparaffinisation and dehydration, respectively. To block endogenous peroxidase, the samples were incubated with 3% hydrogen peroxide. Antigen retrieval was then carried out by heating sections in 10 mM sodium citrate buffer. The samples were blocked with 10% bovine serum albumin and incubated with primary antibodies of CK19 (1:500, Abcam, MA, USA), EpCAM (1:500, Abcam, MA, USA), and Yap1 (1:500, CST, MA, USA) at 4°C overnight. Corresponding secondary antibodies (1:1000, Abcam, MA, USA) were conjugated with horseradish peroxidase and incubated with the sample for 2 h at room temperature. The sections were examined using a fluorescence microscope (Nikon, Japan).

### Quantitative real-time polymerase chain reaction (qRT-PCR)

Total RNA from liver tissue was purified as described previously (Yan et al. 2020). The cDNA was synthesised using a reverse transcriptase PrimeScript 1st Strand cDNA Synthesis Kit (Vazyme, China). PCR amplification was performed using a LightCycler 480 II (Roche, Switzerland) with SYBR Green Supermix (Vazyme), 0.8 μM of each primer, and 1 μL of cDNA. The mRNA levels of connective tissue growth factor (CTGF), cysteine-rich angiogenic inducer 61 (CYR61), c-Myc, CK19, EpCAM, Sox9, and GAPDH were quantified. The primer sequences are listed in Table S1. Relative gene expression changes were calculated using the 2^−ΔΔCT^ method.

### Western blot (WB) analysis

Liver samples were homogenised with a lysis buffer solution (Biotime, Shanghai, China). The homogenates were then used for protein assays with a Bio-Rad Protein Assay Kit (Beyotime, China). Equal amounts of protein samples were subjected to 12% sodium dodecyl sulphate-polyacrylamide gel electrophoresis (SDS-PAGE). The proteins were then transferred onto polyvinylidene fluoride membranes (Millipore, MA, USA). After blocking with 5% non-fat milk, the membranes were incubated with primary antibodies specific for CK19, EpCAM, Yap1 (1:1 000, CST, MA, USA), phospho-large tumour suppressor gene1 (*p*-LATS1) (1:1 000, CST, MA, USA), and GAPDH (1:1 000, CST, MA, USA) overnight at 4°C. The membranes were then exposed to secondary antibodies (1:1 000, Abcam, MA, USA) for 2 h at room temperature and visualised using an enhanced chemiluminescence detection kit (BeyoECL Plus, Beyotime, China).

### Verteporfin treatment of GSD-Ia mice

Five-day-old *G6pc*^−/−^mice were administered verteporfin (10 mg/kg) (MCE, New Jersey, USA) for 15 days, with the wild-type (WT) group receiving no treatment. After the final injection, all mice were sacrificed by cervical dislocation for analysis.

### Statistical analyses

All data were analysed independently based on biological replicates. Statistical analyses were performed using GraphPad Prism v5.0, with *t*-tests used for comparisons between groups and two-way analysis of variance (ANOVA) used for multiple comparisons. A *P*-value of 0.05 was considered statistically significant.

## Results

### Generation of G6pc^−/−^mice by CRISPR/Cas9

**Figure 1.**
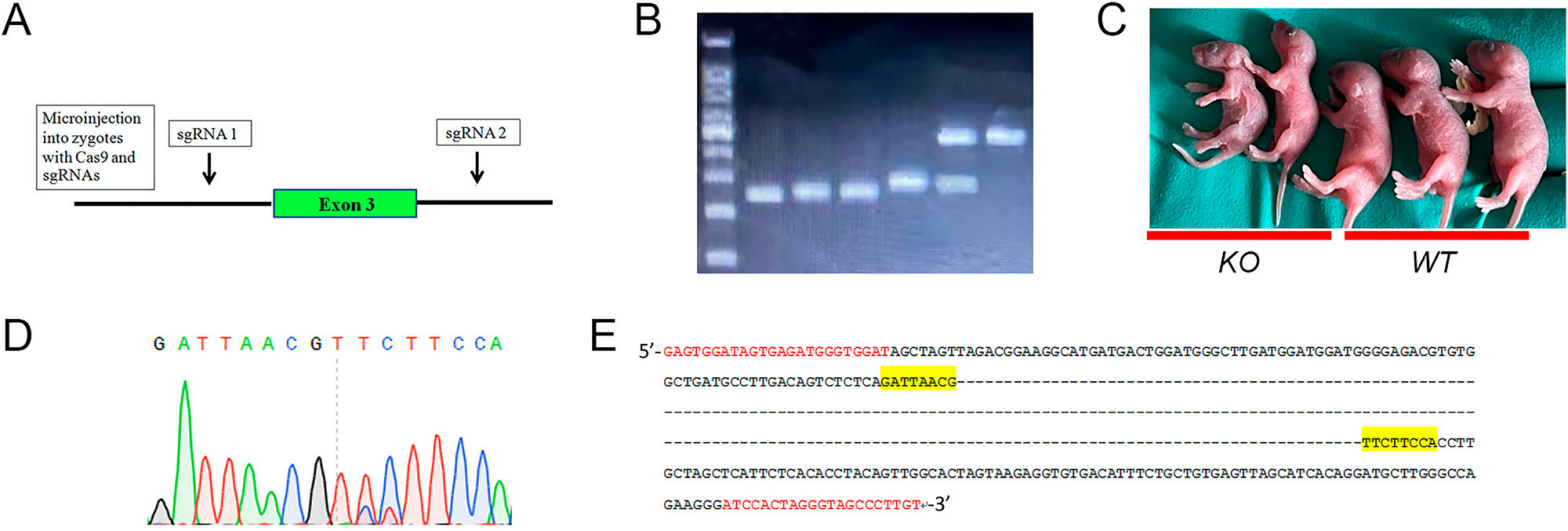
Deletion of exon 3 in *G6pc* gene by CRISPR/Cas9 with two sgRNAs. (A) Overview of targeted *G6pc* exon 3 deletion strategy by microinjection of sgRNA and Cas9 mRNA into mouse zygotes. (B) Genotyping by PCR analysis: ∼450 bp (wild-type, WT), ∼250 bp (*G6pc*^−/−^), and ∼450 and ∼250 bp (*G6pc*^+/−^). (C) Morphology of WT and *G6pc*^−/−^ mice. (D) and (E) Representative sequence results of 215-bp deletion targeting exon 3 of *G6pc* using sgRNA1 and sgRNA2. Primers for sequencing are in red. Yellow indicates sequencing results of D.

Deletion of exon 3 in the *G6pc* gene has been demonstrated to manifest the GSD-Ia phenotype in mice (Lei et al. 1996; Peng et al. 2009; Mutel et al. 2011). To construct a complete *G6pc*^−/−^ mouse model using CRISPR/Cas9, we designed two sgRNAs (sgRNA1 and sgRNA2) to target the introns on either side of exon 3 of the *G6pc* gene (Figure 1(A)). We then co-injected the Cas9 mRNA and *G6pc*-targeting sgRNAs into fertilised eggs derived from WT mice. Two-cell embryos injected with the sgRNAs were then transplanted into recipient oviducts, with the resulting offspring then genotyped by PCR assay (Figure 1(B)). As expected, we observed three PCR bands: i.e. ∼450 bp (WT), ∼250 bp (*G6pc*^−/−^), and ∼450 and ∼250 bp (*G6pc*^+/−^). Based on morphological analysis, the *G6pc*^−/−^ mice were smaller in size than the WT mice (Figure 1(C)). PCR sequencing analysis indicated that the *G6pc*^−/−^ mice lacked the 215-bp sequence containing exon 3 (Figure 1(D,E)). To obtain *G6pc*^−/−^ mice for further study, founder 0 (F0) *G6pc*^+/−^ mosaic mice were crossbred with C57/BL mice to produce F1 *G6pc*^+/−^ mice, and F1 *G6pc*^+/−^ mice were then mated with each other to produce F2 *G6pc*^−/−^mice. Off-target analysis of *G6pc*^−/−^ mice showed no obvious mutations at 10 potential ‘off-target’ sites of the four different sgRNAs (Table S2). Therefore, we generated *G6pc*^−/−^ mice via the CRISPR/Cas9 system. The survival rates of G6pc KO mice were showed in Figure S1.

### Phenotype characterisation of G6pc^−/−^mice

**Figure 2.**
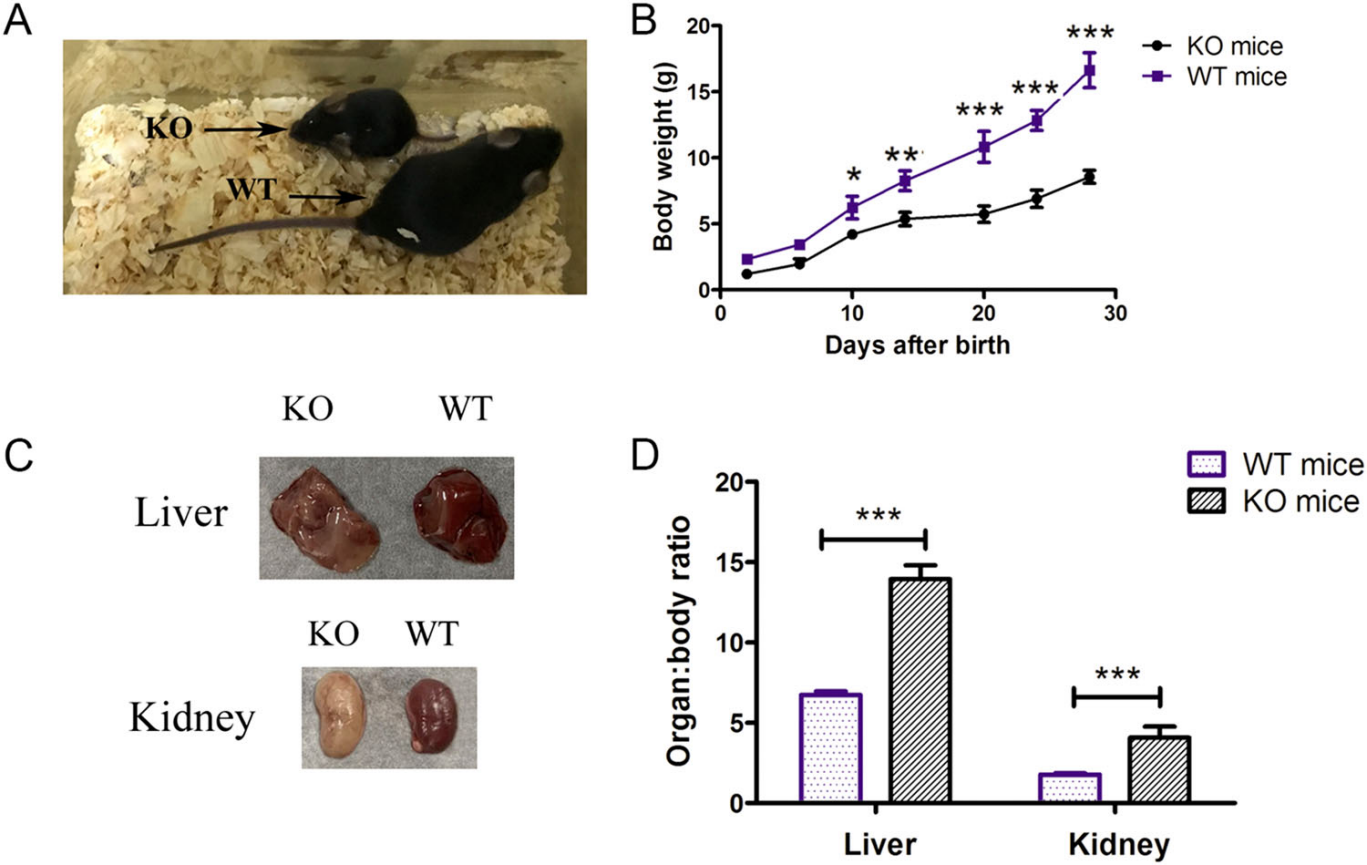
Phenotypic analysis of *G6pc*^−/−^ mice. (A) Morphology of WT and *G6pc*^−/−^ mice. (B) Postnatal development of WT and *G6pc*^−/−^ mice. (C) Liver:body- weight ratio and kidney:body weight ratio in WT and *G6pc*^−/−^ mice. *N* = 8. ***P < 0.001 compared to WT group.

Most *G6pc*^−/−^ mice survived weaning, but very few survived beyond four weeks of age. We evaluated the growth rate of *G6pc*^−/−^ mice from birth to four weeks. Data showed that the weights of the *G6pc*^−/−^ mice were significantly lower than that of the WT mice (Figure 2(A,B)), implying that the *G6pc* knockout mice had slower growth rates than their WT littermates. As *G6pc*^−/−^ mainly results in liver and kidney dysfunction, we measured the liver:body weight ratio and kidney:body weight ratio in 18- to 20-day-old *G6pc*^−/−^and WT mice. Results showed that both ratios were significantly increased in the *G6pc*^−/−^ mice compared to the WT mice (Figure 2(C,D)).

**Figure 3.**
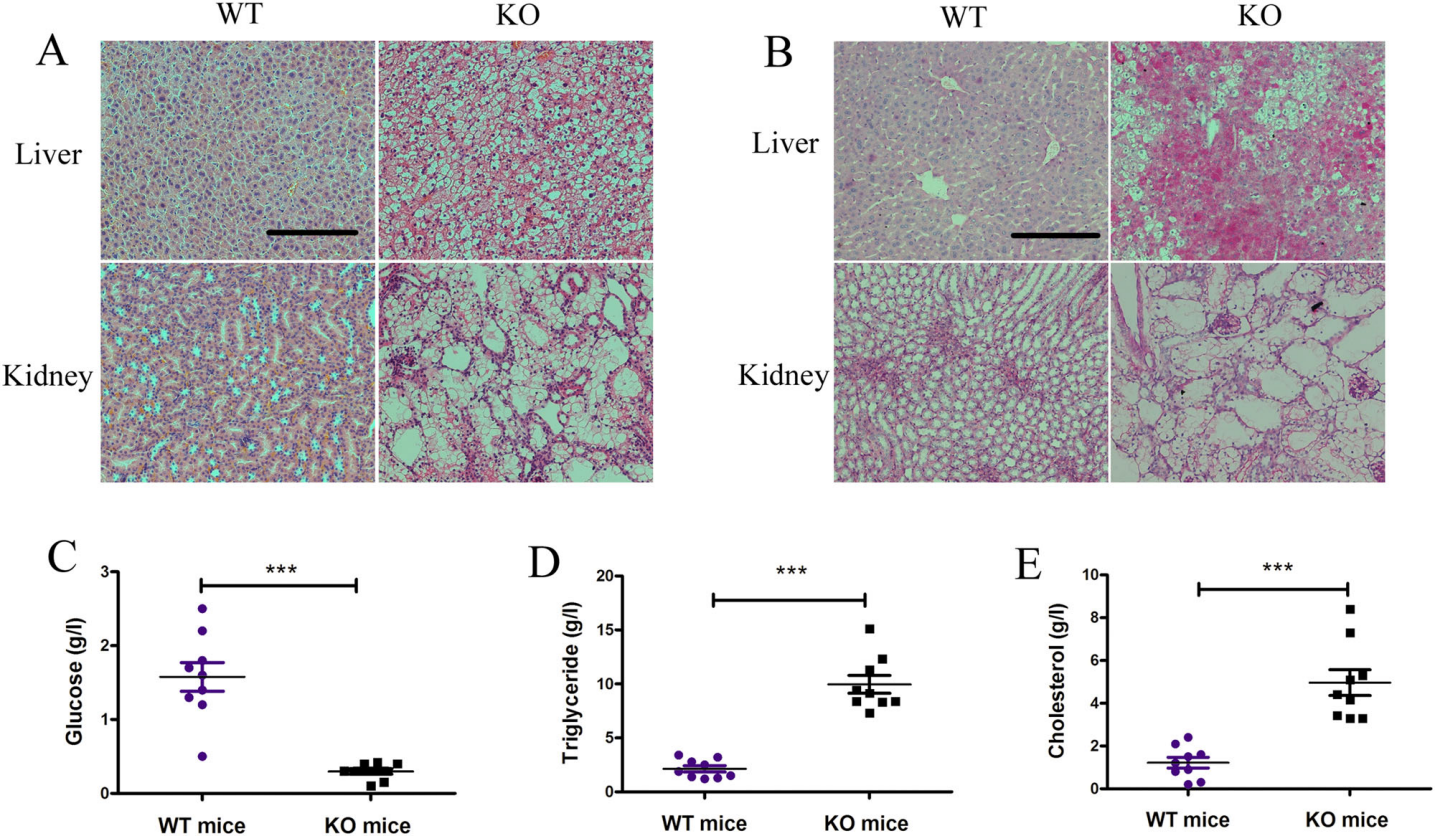
Histological and biochemical analysis of *G6pc*^−/−^ mice. (A) H&E staining showing lipid deposits in liver and kidney tissues of *G6pc*^−/−^ mice. (B) PAS staining showing excess glycogen accumulation in liver of *G6pc*^−/−^ mice. Reduction in glucose level (C) and increase in triglycerides (D) and cholesterol (E) in plasma of *G6pc*^−/−^ mice. Scale bar = 200 μm. *N* = 8. ***P < 0.001 compared to WT mice.

We then analysed histological changes in the liver and kidneys of *G6pc*^−/−^ mice. Consistent with previous studies, we observed lipid deposits in the liver and kidney tissue samples from *G6pc*^−/−^ mice by H&E staining (Figure 3(A)). PAS staining revealed excess glycogen accumulation in the *G6pc*^−/−^ mice compared with that in the WT mice (Figure 3(B)). As expected, we observed a reduction in glucose (Figure 3(C)) and an increase in triglycerides (Figure 3(D)) and cholesterol (Figure 3(E)) in the plasma of *G6pc*^−/−^ mice but did not observe an increase in plasma lactase concentrations. Therefore, *G6pc*^−/−^ mice produced by CRISPR/Cas9 manifested the typical phenotype of human GSD-Ia.

### Induction of ductular reactions in G6pc^−/−^ mice

**Figure 4.**
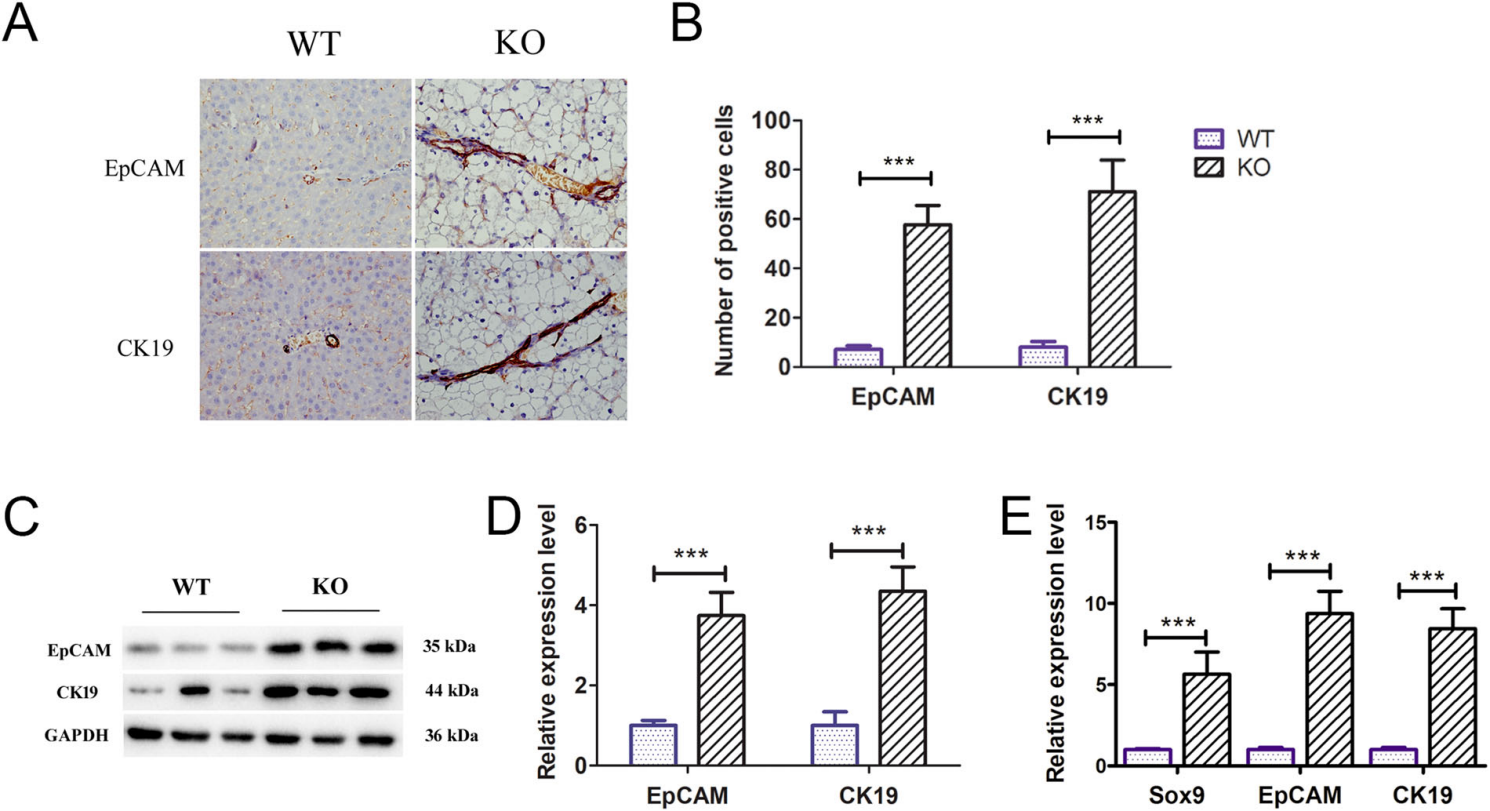
Ductular reaction analysis in *G6pc*^−/−^ mice. (A) Immunohistochemical results revealed that EpCAM-and CK19-positive cells (brown) were significantly increased in *G6pc*^−/−^ mice compared to WT mice. (B) Quantification of positive cell number in A. (C) WB analysis of expression level of ductular reaction markers (EpCAM and CK19) in *G6pc*^−/−^ mice compared to WT mice. (D) Quantification of proteins in C. (E) qRT-PCR analysis of expression levels of ductular reaction markers (Sox9, EpCAM, and CK19) in *G6pc*^−/−^ mice compared to WT mice. Scale bar = 200 μm. *N* = 8. ***P < 0.001 compared to WT mice.

Ductular reactions are an important part of liver repair in hepatic diseases and are typified by reactive cholangiocyte and hepatic progenitor proliferation (Sato et al. 2019a). Previous studies have identified ductular reactions in non-alcoholic fatty liver disease, with this process closely related to the development of HCA and HCC (Richardson et al. 2007; Gadd et al. 2014).Thus, we analysed whether fatty liver induced by *G6pc* deficiency can induce ductular reactions. Ductular reaction cells express biliary proteins such as CK19 and EpCAM. In our study, CK19- and EpCAM-positive cells were clearly observed at the portal-parenchymal interface (Figure 4(A,B)) in *G6pc*^−/−^ mice. As expected, WB analysis revealed that the expression levels of EpCAM and CK19 were significantly increased in *G6pc*^−/−^ mice (Figure 4(C,D)), accompanied by an increase in the expression levels of *CK19*, *EpCAM*, and *Sox9*, as shown by qRT-PCR analysis (Figure 4(E)).Thus, we concluded that the livers in *G6pc*^−/−^mice triggered the activation and proliferation of ductular reaction cells for liver regeneration.

### Yap signalling activity increased in the livers of G6pc^−/−^ mice

**Figure 5.**
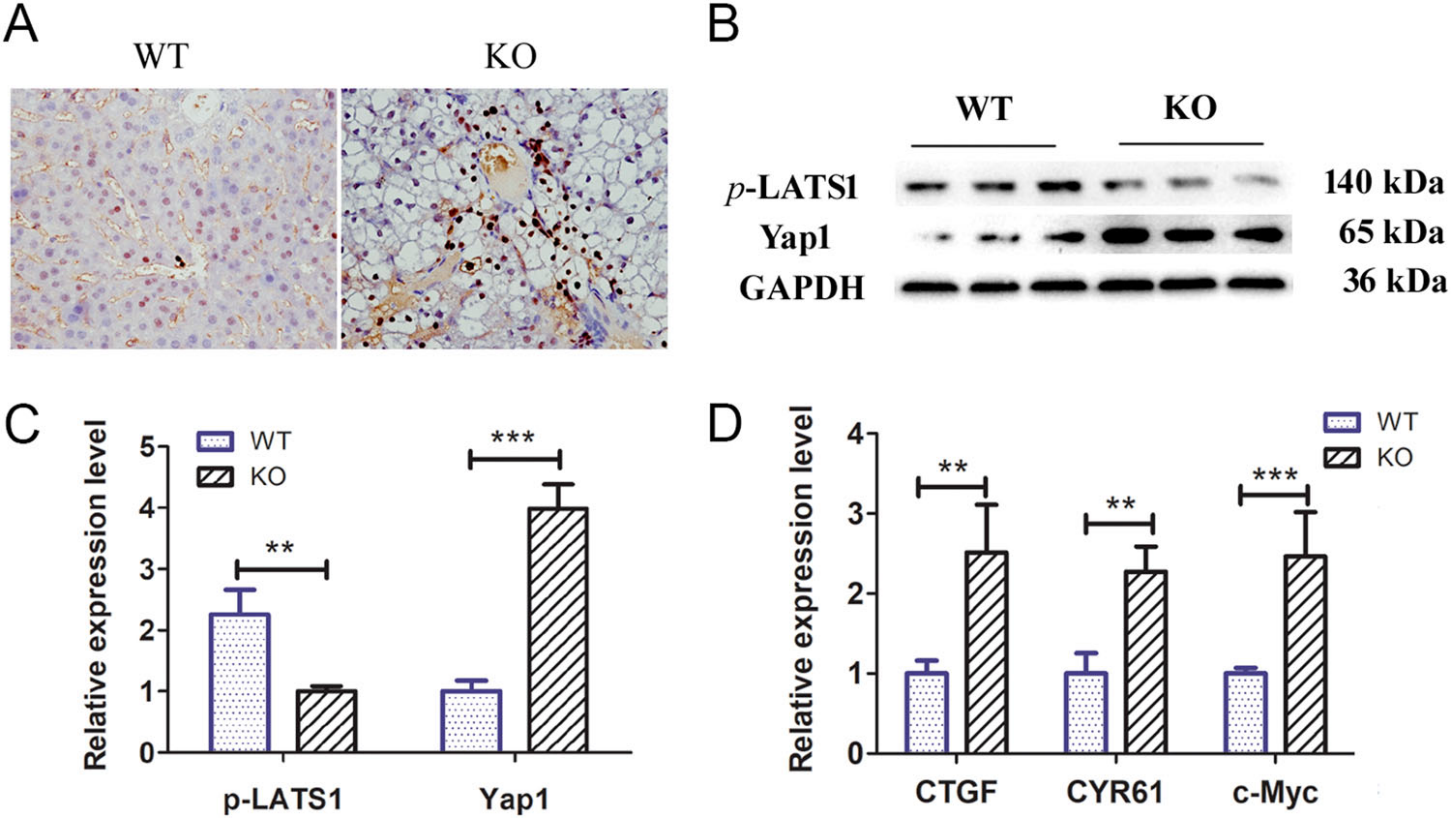
Yap signalling activity analysis in *G6pc*^−/−^ mice. (A) Yap1-positive cells (brown) were observed in ductular reactions in WT group, but rarely expressed in the *G6pc*^−/−^ mice. (B) WB analysis of expression levels of *p*-LATS1and Yap1 in *G6pc*^−/−^ mice compared to WT mice. (C) Quantification of proteins in B. (D) qRT-PCR analysis showed that Yap1 target genes *CTGF*, *CYR61*, and *c-Myc* were significantly increased in *G6pc*^−/−^ mice. Scale bar = 200 μm. *N* = 8. **P < 0.01 and ***P < 0.001 compared to WT group.

Previous studies have shown that Yap signalling activity is positively correlated with the activation of ductular reactions (Jin et al. 2021). As such, we explored Yap signalling pathway activity and target gene expression levels in the livers of 18–20-day-old *G6pc*^−/−^ and WT mice. Immunohistochemical analysis showed significantly higher Yap1-positive cells around the portal vein in the *G6pc*^−/−^ group than in the WT group (Figure 5(A)). WB analysis further indicated that Yap1 expression was increased in the *G6pc*^−/−^ group compared to that in the WT group, while the expression of *p*-LATS1, a negative regulator of Yap1, was decreased (Figure 5(B,C)). As expected, the qRT-PCR results showed that the expression levels of Yap signalling pathway target genes (i.e. *CTGF*, *CYR61*, and *c-Myc*) were significantly increased in *G6pc*^−/−^ mice (Figure 5(D)). These findings indicate that Yap signalling is activated in the mice GSD-Ia model and may be related to activation of hepatic ductular reactions.

### Verteporfin reduces GSD-Ia-induced hepatic ductular reactions

**Figure 6.**
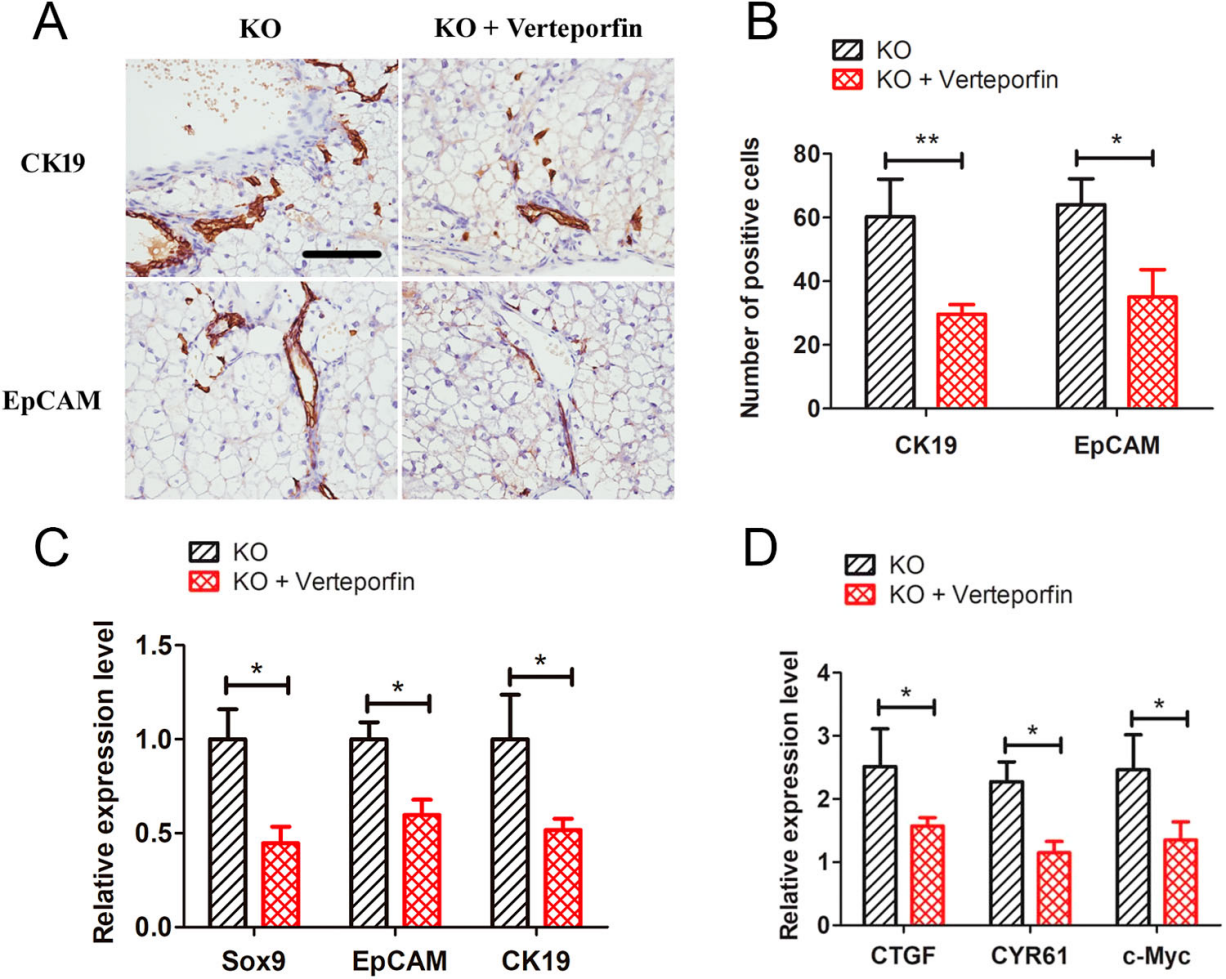
Verteporfin abrogated Yap signalling activity and alleviated ductular reactions in *G6pc*^−/−^ mice. (A) Verteporfin treatment reduced CK19 and EpCAM-positive cells in liver of *G6pc*^−/−^ mice. (B) Quantification of positive cell number in A. (C) qRT-PCR analysis showed that verteporfin treatment reduced expression levels of *CK19*, *EpCAM* and *SOX9*. (D) qRT-PCR analysis showed that Yap1 target genes, *CTGF*, *CYR61*, and *c-Myc*, were significantly decreased in *G6pc* KO mice after verteporfin treatment. Scale bar = 200 μm. *N* = 8. *P < 0.05 and **P < 0.01 compared to WT group.

We next investigated whether inhibition of the Yap signalling pathway could reduce GSD-Ia-induced ductular reactions. Five-day-old *G6pc*^−/−^ mice were treated with 10 mg/kg of verteporfin for 15 days. Results showed that hepatic ductular reactions in the GSD-Ia group were significantly decreased compared with the untreated group, as determined by immunohistochemical staining for CK19 and EpCAM (Figure 6(A,B)). Besides, qRT-PCR analysis also showed that verteporfin treatment reduced expression levels of *CK19*, *EpCAM* and *SOX9* (Figure 6(C)). Further, the expression levels of Yap target genes (i.e. *CTGF*, *CYR61*, and *c-Myc*) were also reduction after verteporfin treatment (Figure 6(D)).

## Discussion

The metabolic disorder GSD-Ia, also known as von Gierke disease, is caused by mutation of the *G6pc* gene and is a characterised by severe hypoglycaemia associated with accumulation of glycogen and fat in the liver and kidneys (Lei et al. 1994; Lu et al. 2016). Mouse models of *G6pc* deficiency closely resemble the phenotype of human GSD-Ia (Lei et al. 1996; Resaz et al. 2019), including hepatocellular adenomas, and therefore are useful models for understanding this disease in humans. Human *G6pc* is a single-copy gene composed of five exons located on chromosome 17q21 (Zhang and Zeng 2016). Mouse *G6pc* also has five exons, with deletion of exon 3 containing the G6Pase-active site Arg-83 usually applied to generate the GSD-Ia model (Lei et al. 1996; Peng et al. 2009; Mutel et al. 2011). Traditional gene editing of specific animals is dependent on ESCs and homologous recombination techniques, which can be time consuming and inefficient.

Consistent with previous studies (Lei et al. 1996; Peng et al. 2009; Mutel et al. 2011), the CRISPR/Cas9-mediated *G6pc*^−/−^ mice exhibited atypical GSD-Ia phenotype, including growth retardation, hypoglycaemia, and high levels of triglycerides and uric acid. In addition, glycogen accumulation and steatosis were observed in the *G6pc*^−/−^ mice. Excessive glycogen and fat accumulation can result in hepatic necrosis and damage, which can induce liver repair/regeneration (Kele et al. 2013).The *G6pc*^−/−^mouse phenotype was consistent with the typical symptoms observed in human GSD-Ia patients, demonstrating successful construction of a GSD-Ia mouse model based on the CRISPR/Cas9 system.

Ductular reactions are initiated as a repair response to biliary and hepatocellular injury (Williams et al. 2014; Planas-Paz et al. 2019). Ductular reactions are a common clinical manifestation of fatty liver, and recent studies have implicated the impact of cholangiocytes and ductular reactions on fatty liver progression (Richardson et al. 2007; Gadd et al. 2014). For instance, fatty liver patients with cholestasis show more advanced histological impairments, including cholangitis, fibrosis, and cirrhosis, compared to age- and sex-matched fatty liver cohorts (Skoien et al. 2013). G6Pase deficiency results in the production of acetyl-CoA via the glycolysis pathway, and acetyl-CoA participates in the biosynthesis of fatty acids and sterols via *de novo* lipogenesis, leading to the development of fatty liver (Qiu et al. 2017). However, whether GSD-Ia patients exhibit the paticductular reactions remains unclear. CRISPR/Cas9 is an efficient gene-editing system, which is used for the generation of gene knockout mice, achieved by frame shift mutations or large fragment deletions (Hwang et al. 2013; Wang et al. 2013). In this study, we deleted exon 3 in the *G6pc* gene with two sgRNAs based on the CRISPR/Cas9 system. The two designed sgRNAs, which targeted the introns on either side of exon 3, efficiently deleted exon 3 and Cas9 mRNA was successfully injected into the fertilised eggs. Sanger sequencing and T7E1 assay did not detect significant off-target activity at the top predicted off-target sites, implying that the sgRNAs specifically targeted the *G6pc* gene. Thus, our results demonstrated the feasibility of generating CRISPR/Cas9-mediated *G6pc*^−/−^ mice, thereby laying the foundation to produce larger animals with *G6pc* knockout using the CRISPR/Cas9 system. Ductular reactions are distinguished by the proliferation of cholangiocytes and hepatic progenitors positive for CK19 and EpCAM (Zhou et al. 2007; Han et al. 2019); thus, we observed the proliferation of cholangiocytes and formation of new bile ducts at the portal-parenchymal interface in *G6pc*^−/−^ mice, which were positive for CK19 and EpCAM. Consistently, the expression levels of CK19, EpCAM, and Sox9 were significantly increased in *G6pc*^−/−^ mice compared to WT mice. Taken together, these results demonstrated the occurrence of ductular reactions in the GSD-Ia mice.

Aberrant Yap signalling activity is implicated in the pathogenesis of chronic liver diseases (Nguyen-Lefebvre et al. 2021). In fact, Yap regulates ductular reactions in pathological livers; for example, conditional knockout of Yap or Yap and Taz in mice models can lead to defects in bile duct morphogenesis and deformed intrahepatic bile ducts (Zhang et al. 2010; Lu et al. 2018). In mice models of cholestatic liver damage induced by bile duct ligation, the reactive proliferation and expansion of bile epithelial cells are dependent on Yap activation, and mice with liver-specific Yap deletion also show a significant reduction in these processes (Bai et al. 2012). Furthermore, Yap is essential for the maintenance of the cholangiocytic phenotype, and a decrease in Yap activation can lead to a reduction in the expression of cholangiocyte markers (Zhang et al. 2010). Our results showed that the expression levels of *p*-LATS1 and Yap1 were significantly decreased and increased in the livers of *G6pc^−/−^* mice, respectively. As the terminal kinase in the Hippo cascade, *p*-LATS1 phosphorylates Yap1, which is then unable to accumulate in the nucleus to regulate gene expression (Driskill and Pan 2021). Based on immunohistochemical staining, we also found that Yap1 was expressed in the cholangiocytes and portal-parenchymal interface cells. However, treatment with verteporfin rescued the ductular reactions and decreased expression of Yap target genes in the *G6pc*^−/−^ mice (Wang et al. 2016). Some studies have demonstrated that verteporfin could inhibit YAP function through up-regulating 14-3-3 sigma sequestering YAP in the cytoplasm (Chao et al. 2015). Moreover, verteporfin could inhibit YAP-induced bladder cancer cell growth and invasion via repressing the target genes’ expression of Hippo signalling pathway (Dong et al. 2018). Therefore, these results suggest that Yap signalling plays an important role in ductular reactions in the liver in GSD-Ia.

Verteporfin, a photosensitizer, which has been used in photodynamic therapy, could inhibit interaction of YAP and TEAD. Moreover, it could block downstream targets of Yap activation (Zhenxue et al. 2019). As above discussion, Yap signalling regulated development of ductular reaction. In our study, administration of verteporfin to mice significantly decreased ductular reaction in the liver in GSD-Ia by inhibition of Yap signalling. Gurda et al study revealed that blocking Yap activity by verteporfin significantly decreased proliferating of cholangiocytes (Gurda et al. 2014). Previous study has reported that about 25% of adult GSD-Ia patient over the age of 25 would develop HCA and HCC. In view of the close relationship between bile duct reaction and the occurrence of HCA and HCC (Jörs et al. 2015). Therefore, our result suggested that verteporfin could be used as a preventive drug for the development of HCA and HCC. In fact, a few studies have reported that verteporfin treatment significantly inhibited HCC development (Gavini et al. 2019; Quan et al. 2021).

Verteporfin treatment didn't change the glucose, TG and Cho biochemical indexes. Besides, we also found that verteporfin treatment did not significantly alter the production of glucose and lipid synthesis. This may be attributed to Yap1 pathway has a limited contribution to glucose and lipid metabolism. Consistent with our study, Song et al. also demonstrated that treatment of HFD-fed mice with verteporfin did not significantly alter the body weight and triglyceride levels (Song et al. 2020).

## Conclusions

In conclusion, we generated complete *G6pc*^−/−^ mice by injection of Cas9 mRNA and two sgRNAs into fertilised zygotes. These *G6pc*^−/−^ mice manifested the typical GSD-Ia phenotype of human patients. In addition, for the first time, we found that ductular reactions occur in GSD-Ia mice, regulated by the Yap signalling pathway. This study not only improves our understanding of GSD-Ia pathophysiology but also highlights the potential use of novel therapeutic approaches for GSD-Ia.
